# Dual trigger with gonadotropin releasing hormone agonist and human chorionic gonadotropin significantly improves live birth rate for women with diminished ovarian reserve

**DOI:** 10.1186/s12958-018-0451-x

**Published:** 2019-01-04

**Authors:** Ming-Huei Lin, Frank Shao-Ying Wu, Yuh-Ming Hwu, Robert Kuo-Kuang Lee, Ryh-Sheng Li, Sheng-Hsiang Li

**Affiliations:** 1 Department of Obstetrics and Gynecology, Mackay Memorial Hospital, Taipei, Taiwan; 2 Department of Medical Research, Mackay Memorial Hospital, Taipei, Taiwan; 3 Mackay Junior College of Medicine, Nursing, and Management, Taipei, Taiwan; 4 Mackay Medical College, New Taipei City, Taiwan; 5 IHMED Fertility Clinic, Taipei, Taiwan; 6 Taipei City Hospital, Heping-Fuyou Branch, Taipei, Taiwan; 7 Department of Obstetrics and Gynecology, Taipei Medical University, Taipei, Taiwan

**Keywords:** Dual trigger, Diminished ovarian reserve, IVF, GnRH agonist

## Abstract

**Background:**

Diminished ovarian reserve (DOR) remains one of the greatest obstacles affecting the chance of a successful live birth after fertility treatment. The present study was set to investigate whether using a “dual trigger” consisted of human chorionic gonadotropin (hCG) plus gonadotropin releasing hormone agonist (GnRH-a) for final oocyte maturation could improve the IVF cycle outcomes for patients with diminished ovarian reserve.

**Methods:**

A total of 427 completed GnRH-antagonist downregulated IVF cycles with fresh embryo transfer (ET) were included in this retrospective analysis. DOR was defined as antral follicle count ≤5 and serum anti-Müllerian hormone level ≤ 1.1 ng/mL. The control group (*n* = 130) used a 6500 IU of recombinant hCG for trigger, and the study group (*n* = 297) used 0.2 mg of triptorelin plus 6500 IU of recombinant hCG for trigger.

**Results:**

The dual-trigger group had significantly higher oocyte fertilization rate (73.1% vs. 58.6%), clinical pregnancy rate (33.0% vs. 20.7%) and live birth rate (26.9% vs. 14.5%) when compared to the hCG trigger group. In addition, the abortion rate (17.4% vs. 37.0%) and embryo transfer cancellation rate (6.1% vs. 15.4%) were both significantly lower in the dual trigger group. The primary outcome measure was the live birth rate per oocyte retrieval cycle. Secondary outcome measures were embryo transfer cancellation rate, clinical pregnancy rate, implantation rate, chemical pregnancy rate and abortion rate per oocyte retrieval cycle.

**Conclusions:**

Dual triggering the final oocyte maturation with GnRH-a and standard dose of hCG can significantly improve the live birth rate, clinical pregnancy rate, and fertilization rate in women with diminished ovarian reserve undergoing GnRH antagonist down-regulated IVF-ICSI cycles.

## Background

The management of the “diminished ovarian reserve” (DOR) or poor responder to ovarian stimulation remains one of the most challenging aspects in the field of fertility treatment. According to the data published by Society for Assisted Reproductive Technologies, the number of women aged 40 years or older seeking fertility treatment had increased by more than 80% between 1999 and 2008 [1]. Therefore, it came as no surprise that the population of poor responders has grown exponentially over the years. It has been estimated of all couples treated in IVF units, 10–24% of them were poor responders [2].

In attempts to optimize IVF cycle outcomes for poor responders, modifying the steps of conventional ovarian stimulation protocols have been proposed, such as different luteal phase pretreatments, ovarian stimulation protocols, as well as addition of various supplements. Though most of the modifications had limited success, an optimal protocol for poor responders has remained elusive [3].

Nevertheless, one of the most critical steps during IVF treatment has remained mostly untouched, which is the use of human chorionic gonadotropin (hCG) for triggering final oocyte maturation before retrieval. The discovery of hCG was made by Ascheim and Zondek nearly ninety years ago [4]. From the blood and urine of pregnant women, they extracted an injectable gonad-stimulating substance that could induce follicular maturation and ovarian stromal luteinization in immature mice. Until now, administering 5000 IU to 10,000 IU of hCG 34–36 h prior to oocyte retrieval still remained as the standard protocol for the induction of final oocyte maturation in IVF cycles worldwide.

As an effort to eliminate the incidence of ovarian hyperstimulation (OHSS), substitution of hCG with gonadotropin-releasing hormone agonist (GnRH-a) for trigger was proposed for IVF high responders [5, 6]. Triggering with GnRH-a indeed have minimized the risk of OHSS, but the pregnancy rate was found to be adversely affected due to the impaired luteal function [5, 6]. In order to rescue the luteal phase, strategies such as aggressive post-retrieval progesterone and estrogen supplementation [7], or adding a reduced dose of hCG either at oocyte retrieval [8] or intermittently during luteal phase [9] were implemented. Then the concept of “dual trigger” with GnRH-a plus a reduced dose of hCG emerged as another mean to correct the dysfunctional luteal phase after GnRH-a triggering. In a study consisted of high responders, Shapiro et al. first demonstrated that acceptable rates of fertilization, implantation, clinical pregnancy, ongoing pregnancy, and early pregnancy loss could still be obtained using a dual trigger regimen consisted of 4 mg leuprolide acetate and 1000 to 2500 IU of hCG [10]. In two follow-up studies on high responders, the group that used dual trigger consistently showed superior ongoing pregnancy rate [11] and live birth rate [12] when compared to the group that used GnRH-a trigger alone. Most importantly, the use of dual trigger was not associated with the increased incidence of OHSS [10–12].

In two similar studies consisted of normal responders undergoing IVF, significant improvement in total number of retrieved oocytes [13], number of mature (MII) oocytes [13], rates of embryo implantation [13], clinical pregnancy [13, 14], ongoing pregnancy [14] and live birth [13] were also observed when dual trigger was used instead of hCG trigger. These findings further indicated that in addition to reducing the incidence of OHSS, the GnRH-a component in the dual trigger could also have important roles in oocytes maturation and embryo implantation.

In light of the promising results demonstrated for the high and normal responder groups, the present study is set to investigate whether dual trigger with standard dose hCG and GnRH-a can also have a role in improving the IVF cycle outcomes among patients of diminished ovarian reserve.

## Methods

### Study design

This is a retrospective cohort analysis consisted of GnRH antagonist IVF cycles from October, 2013 through December, 2016 at the Infertility Division of Mackay Memorial Hospital in Taipei City, Taiwan. The study protocol was approved by the institutional review board of Mackay Memorial Hospital.

### Study participants

In the present study, diminished ovarian reserve was defined as the presence of both serum anti-*Müllerian* hormone level (≤ 1.1 ng/mL) and low antral follicle count (≤ 5) at the time of initiation of ovarian stimulation. Exclusion criteria were co-existing endocrine disorders (diabetes mellitus, hyperprolactinemia, thyroid dysfunction, congenital adrenal hyperplasia, Cushing syndrome), untreated hydrosalpinx, and uterine anomaly confirmed either by hysterosalpingography or hysteroscopy. After applying the exclusion criteria, a total of 427 IVF cycles with fresh embryo transfer (ET) were included for final analysis (hCG trigger/control group: *n* = 130; dual trigger/study group: *n* = 297).

### Ovarian stimulation protocols

After a baseline pelvic ultrasound, controlled ovarian stimulation was commenced on the third day of the menstrual cycle for three consecutive days with a fixed starting dose of r-FSH (Gonal-F; Merck Serono Biopharma) of 450 IU for all subjects. The starting dose was chosen based on observations from previous studies [15, 16] that demonstrated no added benefit to daily gonadotropin dosing above 450 IU. The daily r-FSH dose was then adjusted according to ovarian response. When at least one follicle reached 14 mm in diameter, GnRH antagonist (Cetrotide; Merck Serono Biopharma) was added to the stimulation protocol. When at least two leading follicles reached 18 mm in diameter, induction of final oocyte maturation was triggered either by 6500 IU of recombinant hCG (Ovidrel; Merck Serono Biopharma), or by a combination of 6500 IU of recombinant hCG and 0.2 mg of triptorelin (Decapeptyl; Ferring Pharmaceuticals). The choice of triggering method was based on the attending physicians’ discretion.

Oocyte retrievals were performed under transvaginal ultrasound guidance, 35 to 36 h post triggering. Standard insemination procedures were performed for all cases, with exceptions in cases with male factor, for which intracytoplasmic sperm injections (ICSI) were performed instead. All embryo transfers were performed on day-3 after oocyte retrieval.

### Luteal phase support and confirmation of pregnancy

Luteal phase supplement with daily vaginal progesterone gel (Crinone 8%; Merck Serono Biopharma) was initiated on the day of oocyte retrieval. Serum beta-hCG level was measured 14 days post oocyte retrieval, and a value above 5 IU/mL was considered as positive pregnancy. The luteal phase support was then continued until the 10th weeks of gestation for all positive pregnancies.

### Outcome measures

The primary outcome measure was the live birth rate per oocyte retrieval cycle. Secondary measures were embryo transfer cancellation rate, clinical pregnancy rate, implantation rate, chemical pregnancy rate, and abortion rate per oocyte retrieval cycle. Embryo transfer cancellation was defined as discontinuation of embryo transfer due to fertilization failure or embryonic cleavage arrest. Live birth was defined as delivery of a viable fetus of > 23 weeks of gestation. Clinical pregnancy was defined as the visualization of the fetal heart beat by ultrasound between the 5th to 6th weeks of gestation. The implantation rate was calculated as the total number of observed gestational sacs divided by the total number of transferred embryos. Biochemical pregnancy was defined as a transient positive serum beta-hCG level without subsequent development of visible gestational sac. Abortion was defined as blighted ovum or fetal demise before 20th weeks of gestation.

### Statistical analysis

The statistical analysis was performed using MedCalc 10.2 (MedCalc Software, Mariakerke, Belgium). Continuous variables were presented as mean with standard deviations (SD), and the between-group differences were analyzed by independent Student’s t-test. Categorical variables were presented as raw frequencies with corresponding percentages, and the between-group differences were analyzed either by Chi-square test with Yates correction if indicated, or by Fisher exact test. A *P*-value < 0.05 was considered as statistically significant.

## Results

The baseline characteristics for the control and the study group are summarized in Table 1. The was no significant differences in the patient age, serum AMH level, and etiologies of infertility. The response to ovarian stimulation and cycle outcomes are presented in Table 2. There was no significant difference in the total r-FSH dose, duration of stimulation, endometrial thickness, and hCG day serum hormone profiles between the control and the study group. The mean number of total retrieved oocytes and mature metaphase II (MII) oocytes were also similar between the two groups (Table 2). In terms of cycle outcome, the dual-trigger group demonstrated a significantly higher fertilization rate (73.1% vs.58.6%; *P* = .015), clinical pregnancy rate (33.0% vs. 20.7%, *P* = .035) and live birth rate (26.9% vs. 14.5%, *P* = .014) compared to the hCG trigger group (Table 3). The cycle cancellation rate (6.1% vs. 15.4%, *P* = .003) and abortion rate were significantly lower in the dual trigger group (17.4% vs.37.0%, *P* = .037). There was no incidence of OHSS in either group.

**Table 1:** Comparison between hCG and dual-trigger protocols: Patient demographics

Patients ‘baseline profiles	Control group (hCG)	Study group (hCG + triptorelin)	*P* value
Total number of egg retrieval cycles	130	297	–
Age (y)	38.26 ± 3.27	37.85 ± 3.73	NS
AMH (ng/ml)	0.81 ± 0.26	0.84 ± 0.23	NS
Etiology of infertility (%)			
Male Factor	37.1	28.8	NS
Tubal Factor	30.7	29.6	NS
Ovulation Dysfunction	51.3	50.4	NS
Endometriosis	41.6	41.2	NS
Uterine factor	2.2	4.4	NS
Unexplained	10.5	4.5	NS

Note: Values are expressed as mean ± standard deviation or percentage. *AMH* anti-*Müllerian* hormone, *hCG* human chorionic gonadotropin, *NS* non-statistically significant

**Table 2:** Comparison between hCG and dual-trigger protocols: Outcomes of ovarian stimulations

	Control group (hCG)	Study group (hCG + triptorelin)	*P* value
E_2_ on trigger day (pg/ml)	775.29 ± 500.1	628.16 ± 503.4	NS
No. of oocytes Retrieved	3.40 ± 1.36	3.27 ± 1.53	NS
No. of MII oocytes Retrieved	2.85 ± 1.33	2.75 ± 1.42	NS
Fertilization rate (%)	58.63 ± 36.23	73.10 ± 71.80	0.015
No. of embryo transferred	1.67 ± 1.10	2.06 ± 1.11	NS

Note: Values are expressed as mean ± standard deviation or percentage. *E*_2_ estradiol, *MII* metaphase II, *hCG* human chorionic gonadotropin, *NS* non-statistically significant

**Table 3:** Comparison between hCG and dual-trigger protocols: Outcomes of IVF-ICSI cycles

	Control group (hCG)	Study group (hCG + triptorelin)	*P* value	
Cancellation of embryo transfer (%)	15.4 (20/130)	6.1 (18/297)	0.003	
Implantation Rate (%)	10.58 ± 2.45	15.02 ± 1.74	NS	
Biochemical pregnancy rate per cycle (%)	4.6 (6/130)	2.0 (6/297)	NS	
Clinical pregnancy rate per cycle (%)	20.7 (27/130)	33.0 (92/297)	0.035	
Live birth rate per cycle (%)	13.1 (17/130)	27.2 (76/297)	0.014	
Abortion rate (%)	37.0 (10/27)	17.4 (16/92)	0.037	
Twin pregnancy rate (%)	1.5 (2/130)	4.4 (13/297)	NS	

Note: Values are expressed as mean ± standard deviation or percentage. *hCG* human chorionic gonadotropin, *NS* non-statistically significant

## Discussion

The present study offered a novel strategy for improving the outcomes in patients with diminished ovarian reserve undergoing GnRH antagonist down-regulated IVF-ICSI cycles. By triggering the final oocyte maturation with a combination of standard dose hCG and GnRH-a, the study group demonstrated a significantly higher oocyte fertilization rate, clinical pregnancy rate and live birth rate, while incurring a lower embryo transfer cancellation rate and abortion rate when compared to the control group. The observed benefits of using the dual-trigger concurred with previous reports [17–19], although the data on the live birth rate was not provided in those studies. To the best of our knowledge, this is the first ever study that demonstrated the positive effects of dual triggering in diminished ovarian reserve population in terms of improved live birth rate.

Ever since its inauguration in 2011, the Bologna Criteria has been one of most adopted guidelines for defining poor responders [20]. Nevertheless, the above classification is not without limitation. For example, in a previous report that showed that dual trigger significantly increased the number of oocytes retrieved and the oocyte maturation rate in poor responders, the authors only recruited the subjects that fulfilled the Bologna criteria [18]. By applying such arbitrary outline, other “potential” poor responders who were under 40 years of age, as well as ones without previous IVF stimulation cycles would had been excluded. In the present study, we used diminished ovarian reserve tests (AMH < 1.1 ng/ml and AFC < 5) as our primary inclusion criteria. Thus, more patients with low oocytes yield could be included in our analysis, which enable the results derived from the study to be applicable to broader populations and clinical settings.

One of the advantages of triggering with GnRH-a is the simultaneous induction of a mid-cycle FSH surge that is similar to the hormonal events in a natural ovulatory cycle. Animal studies have confirmed the importance of FSH in up regulating of luteinizing hormone (LH) receptor sites formations in granulosa cells [21, 22]. The expression of LH receptors is essential for preparing the maturing follicle for the pre-ovulatory LH surge and the luteinzation of granulosa cells. FSH also has a key role in promoting the resumption of oocyte meiosis [23, 24] and the expansion of cumulus cells [25, 26], all of which are critical steps in the oocyte maturation process. Therefore, one of the proposed benefits of GnRH-a triggering is the increased rate of mature oocytes retrieved. From the studies focusing on high responders [5, 8] normal responders [13], and poor responder (18), the GnRH-a trigger group had consistently resulted in higher proportion of metaphase II (MII) oocytes at the time of retrieval compared to the hCG group. In a related study by Griffin et al., patients with a history of > 25% of immature oocytes at previous retrieval had marked improvement of oocyte maturity when dual-triggered with standard dose hCG and GnRH-a in subsequent IVF cycles [27]. Though similar effects of improved oocyte maturation was not observed in the current study, this was not unexpected since low oocyte yield was anticipated for this specific study population, and a statistically significant difference would have been difficult to observe with the present sample size. Nevertheless, the trend toward improved implantation and reduced chemical pregnancy rates, as well as significantly higher rates of oocyte fertilization, clinical pregnancy and live birth showed in the present study could also be viewed as an enhancement of oocyte competence from dual triggering. In a related study, the authors simulated an artificial mid-cycle FSH surge by adding a single bolus of FSH (450 IU) to the hCG as trigger [28]. Compared to the control group triggered by hCG and placebo, the study group triggered by FSH and hCG had significantly improved oocyte competence, as demonstrated by greater oocyte recovery and fertilization rate. In another report, a case of repetitive immature oocytes and empty follicle syndrome was also successfully treated with dual triggering, resulting in a singleton live birth at term [29].

Furthermore, in a pilot study by Haas et al., the differential messenger RNA (mRNA) expression of reproduction-related genes in the oocyte granulosa cells (GCs) of patients triggered with hCG were compared to the same cohorts triggered with GnRH-a plus hCG (dual trigger) in the subsequent IVF cycles [30]. The authors found that higher levels of amphiregulin and epiregulin were expressed in the GCs after dual triggering. Amphiregulin and epiregulin are ligands of the epidermal growth factor (EGF) receptors, and both have been indicated to participate important roles in cumulus expansion [31, 32], oocytes maturation [32], and meiosis resumption [31]. Since both amphiregulin and epiregulin expressions are up-regulated directly in the presence of FSH and LH, the surges of these two hormone induced by the GnRH-a trigger may be one of the mechanisms responsible for the improved fertilization, clinical pregnancy rate and live birth rate observed in the present study.

Interestingly, Haas et al. had also reported a modified “double trigger” protocol aimed for patients with low proportion of mature oocytes per follicle in the preceding IVF cycles [19, 33]. Specifically, the GnRH-a and hCG was administered 6 h apart, at 40 and 34 h prior to oocyte retrieval, respectively. When compared to previous cycles triggered with hCG alone, the subjects in both studies showed significant higher number of retrieved oocyte, 2PN embryos, available embryo for transfer, and ongoing pregnancy rate when modified trigger was used. Though the two studies contained very small sample sizes (*n* = 8; *n* = 12), their results also indicated improvement in oocyte and embryo quality when GnRH-a was included as part of trigger regimen along with hCG. In addition to further affirm the observations made from the present study, these results also showed that the GnRH-a component can either be administered simultaneously with hCG or preceding it without losing its positive effects. A summary of all the reported GnRH-a and hCG trigger combinations are provided in the comprehensive review by Orvieto [34]. More importantly, the article is one of the first to provide an individualized patient-based approach in choosing the most suitable trigger regimen for different subgroups of infertility patients, which not only can optimize the treatment outcomes but also minimize the risks of OHSS.

In addition, another proposed advantage with dual trigger is potential enhancement of endometrial receptivity by the GnRH-a component. Significant elevation of both isoforms of human GnRH mRNA expression have been detected in the secretory phase of the human menstrual cycle [35–37], indicating the possible role of these hormones in regulation of endometrial receptivity [35, 38]. Specifically, in vitro studies with human extra-villous cytotrophoblasts and decidual stroma cells have demonstrated the ability of GnRH to activate urokinase type plasminogen activator, a key component in decidualization and trophoblast invasion [39, 40]. Therefore, inclusion of GnRH-a as part of luteal support regimen has been explored as a mean to improve the implantation rate. In a related meta-analysis [41], the authors concluded that a single-dose of GnRH-a in the luteal phase could significantly enhance the implantation rates in either GnRH-a or GnRH-antagonist down-regulated ICSI cycles. Interestingly, improvement in clinical and ongoing pregnancy rates with luteal GnRH-a were only observed in GnRH antagonist downregulated cycles. Though the dual trigger in the present study was administered in the pre-ovulation period, we believe with the strong affinity of GnRH-a to its receptors as well as its longer half-lives, GnRH-a could still exert its positive effects during embryo implantation period. In the previous studies consisted of normal responders, the dual-riggered group had consistently demonstrated higher implantation rates when compared to conventional hCG triggered group [13, 14]. Schacter et al. postulated the positive effect of pre-ovulatory GnRH-a administration on embryo implantation could be attributed to displacement of the GnRH-ant from the endometrial GnRH receptors by the GnRH-a, thus enabling proper post-receptor actions for implantation [14].

With evolving clinical acceptance and understanding, triggering with GnRH-a has since become an integral part of modern IVF practices, especially in high responders, oocytes donors, as well as oncology patients seeking fertility preservation. Nevertheless, other than the known hypothalamic hypogonadism patients, there remains a subsets infertile patients who are not suitable candidates for triggering with GnRH-a alone. In a retrospective analysis by Meyers et al., the overall incidence of suboptimal response to GnRH-a trigger was reported to be 5.2%, with long term oral contraceptive use and low serum LH level (≤ 0.5 mIU/mL) on the day of triggering identified as the most relevant risk factors [42]. Specifically, suboptimal response was defined as an inadequate hormone surge with low serum LH level (< 15 mIU) drawn 8–12 h after ovulatory trigger. All subjects were young age, presumed normal responders triggered by either GnRH-a alone or by dual trigger (low-dose hCG (1000–3300 IU) plus GnRH-a). In contrast to the previous reports by the Humaidan group (8), the rationale of adding a low dose hCG was intended as an alternative trigger rather than provide luteal support. Since the rate of suboptimal response to the GnRH-a trigger reported was 5.2% [42], it seemed the hCG component in the dual trigger can at least partially compensate the inadequate response to GnRH-a induced hormone surge.

From the above data, we proposed the GnRH-a component in the dual trigger has two distinct yet related functions. In an elegant study by Fauser et al., the authors demonstrated that the GnRH-a trigger would induced a rapid LH and FSH surge at 4 h and 8 h post injection, respectively [43]. Therefore, the area under curve (AUC) of serum FSH and LH concentrations within the first 24 h for oocytes maturation would be much higher from the GnRH-a trigger compared to the hCG trigger. As a result, dual trigger may impose an earlier and more potent oocyte maturation effects than hCG trigger alone within first 24 h. Since the LH surge induced by GnRH-a occurs more rapidly than the hCG triggering peak 4 h vs. 12–24 h) [43], the hCG component of the dual trigger could serve as an second / rescue trigger in the event of poor response to GnRH-a as described in the report by Myers et al. [42]. Secondly, the bolus of hCG could also correct the defective luteal phase resulted from the GnRH-a trigger, as described in the reports 8). Though the timing and dosing of hCG administration in the present study were different from the reports by Humaidan group [8], the rationale of utilizing hCG as part of enhanced luteal support was essentially the same.

In conclusion, the results from the present study demonstrated that in GnRH antagonist down-regulated IVF-ICSI cycles, dual triggering the final oocyte maturation with GnRH-a and standard dose of hCG could significantly improve the rate of fertilization, clinical pregnancy as well as live birth in women of diminished ovarian reserve. Furthermore, the benefit of lowered cycle cancellation rate would also enable greater percentage of patients with diminished ovarian reserve to reach the final stage of their IVF treatment, thereby enhancing their chance of achieving a successful pregnancy as well as alleviating the psychological stress. The limitation of the present study is its retrospective design. Future prospective randomized controlled studies, though difficult, are still required to affirm the beneficial effects of dual triggering in diminished ovarian reserve. Nevertheless, the data from the present study concurred with other studies of dual triggering, which further confirmed the proof-of-concept that calls for a possible paradigm shift in ovulation-triggering agent for GnRH-antagonist cycles.

## Abbreviations

FSH: Follicular stimulating hormone
GnRH: Gonadotropin releasing hormone
hCG: Human chorionic gonadotropin
ICSI: Intracytoplasmic sperm injection
IVF: In vitro fertilization
OHSS: Ovarian hyperstimulation syndrome
